# Functional Food Based on Potato

**DOI:** 10.3390/foods12112145

**Published:** 2023-05-26

**Authors:** Jian Xu, Yang Li, Lovedeep Kaur, Jaspreet Singh, Fankui Zeng

**Affiliations:** 1 Research & Development Center for Eco-Material and Eco-Chemistry, Lanzhou Institute of Chemical Physics, Chinese Academy of Sciences, Lanzhou 730000, China; xujian1980@licp.cas.cn (J.X.); m15214046636@163.com (Y.L.); 2 Riddet Institute, School of Food and Advanced Technology, Massey University, Palmerston North 4442, New Zealand; l.kaur@massey.ac.nz (L.K.); j.x.singh@massey.ac.nz (J.S.)

**Keywords:** starchy food, potato protease inhibitors, phytochemicals, antioxidants, cancer prevention

## Abstract

Potato (*Solanum tuberosum* L.) has gradually become a stable food worldwide since it can be a practical nutritional supplement and antioxidant as well as an energy provider for human beings. Financially and nutritionally, the cultivation and utility of potatoes is worthy of attention from the world. Exploring the functionality and maximizing the utilization of its component parts as well as developing new products based on the potato is still an ongoing issue. To maximize the benefits of potato and induce new high-value products while avoiding unfavorable properties of the crop has been a growing trend in food and medical areas. This review intends to summarize the factors that influence changes in the key functional components of potatoes and to discuss the focus of referenced literature which may require further research efforts. Next, it summarizes the application of the latest commercial products and potential value of components existing in potato. In particular, there are several main tasks for future potato research: preparing starchy foods for special groups of people and developing fiber-rich products to supply dietary fiber intake, manufacturing bio-friendly and specific design films/coatings in the packaging industry, extracting bioactive proteins and potato protease inhibitors with high biological activity, and continuing to build and examine the health benefits of new commercial products based on potato protein. Notably, preservation methods play a key role in the phytochemical content left in foods, and potato performs superiorly to many common vegetables when meeting the demands of daily mineral intake and alleviating mineral deficiencies.

## 1. Introduction

The role of potatoes on the table varies with the development of the region. In many developed countries, it acts as a vegetable, with intakes varying from the lowest value in the UK of 102 g to the maximum intake in Belarus of 181 g per capita per day for adults [1]. On the other hand, especially in some rural areas of America and in the highlands of Latin American countries, the daily consumption of potato by adults is as much as 5–6 times the quantities in developed countries [2]. China ranked first in potato production and made up about 24.53% of the world production in 2018, according to the Food and Agriculture Organization of the United Nations database [3]. The potatoes have gained fame as a globally consumed food crop mostly due to its qualities of tremendous yield per unit area [4], affordability [5], and large daily consumption. Potato functions as an antioxidant, antibacterial, anti-inflammatory, anti-obesity, anti-cancer, and anti-diabetes product in human and animal clinical studies [6]. Compounds existing in potatoes such as starch, protein, fiber, mineral, polyphenols, and carotenoids are thought to have a variety of benefits for human beings [6], although there are significant differences in the nutritional profiles and contributions of different potato cultivars to the human body. For the whole potato tuber, the carotenoid concentration of yellow-fleshed potatoes is higher than that of white- or purple-fleshed potatoes, while the anthocyanin concentration of purple potatoes is higher than that of red- or white-fleshed potatoes. Since polyphenols are mainly concentrated in the peel, colored potatoes generally have higher anthocyanin and carotenoid concentrations than whole white potatoes. The potato’s beneficial properties are attributed to the presence of these nutritional compositions.

However, with the increasing concern about weight and diabetes as well as cancer, potato as a carbohydrate-rich food is generally considered to have a high glycemic index and glycemic load [7,8], which is routinely described is being related to the risk of type 2-diabetes [8,9] and weight gain [10]. Promisingly, some studies support the positive effects of eating potatoes on our overall health [11], even though a small minority of them claim that the consumption does not affect weight control or diabetes [6]. In contrast, some research indicates that the consumption of potatoes has a direct association with an increase in hypertension [12] since potassium supplementation has a potential preventative effect on hypertension and chronic disease [13]. Hypertension is a major risk factor for cardiovascular diseases, especially coronary heart disease, stroke, and heart failure as well as renal failure (WHO, 2004). This is the reason for the decline in the consumption of potatoes in the past few decades. To change the negative trend and alleviate grain shortages by eating potatoes in some areas, it is crucial to make clear the functions of nutritionally important components of potato tubers and what factors should be first considered when processing certain potatoes.

Prior to this review, there have been a number of studies discussing the functional properties of potatoes, but they lack conclusive results about the main factors of those compounds. Thus, this review intends to summarize the factors that influence the changes in the main functional components of potatoes and discuss the focus of referenced literature, with the aim to help to make it clear which areas require further research efforts. This recent research has revealed possible directions for breeding programs and processing approaches to improve the functional composition of potatoes.

## 2. Potato Starch and Its Functional Properties

### 2.1. Structure of Potato Starch

Starch is the main carbohydrate reserved for providing energy in potato tubers [14] and is a semi-crystalline biopolymer that contains two major components: amylose and amylopectin. Native potato starch contains a large proportion (nearly up to 70%) of amylopectin. Amylose, containing about 0.2% α-(1→6) linkages, is a long linear glucan, with the molecular chain turning every six glucosyl units to form a helical structure [15] which can form a double helix by associating with another glucan chain. The established double helix is resistant to digestive enzymes [16], and therefore the proportion of amylase in starch affects starch digestion. Amylopectin, containing approximately 5% of α-(1→6) linkages, exists as a complex branch structure and therefore is less likely to crosslink with other molecules. Thus, starch with a higher ratio of amylose to amylopectin is prone to form gels than starch with a lower ratio, which is usually glue-like after heat–moisture treatment [17].

Based on the structure of the two types of starch, starch with a high amount of amylase is not recommended in complementary feeding [17], but “high amylose” crops have been widely prevailing in the health food markets because high-amylose starch can enhance colonic fermentation, acting similarly to dietary fiber [18]. The properties of starch, to some degree, depends on the ratio of amylose and amylopectin, and they are also affected by the shape and size of starch granules. The utility and development of starch involve not only food processing but also paper, cosmetic, textile industries, etc. [19].

### 2.2. Digestive Properties of Potato Starch

The cooking process and storage conditions can greatly influence the molecular properties, functional properties, and digestibility of starch. For instance, uncooked potatoes are resistant to digestion, whereas cooked potatoes can be easily digested. Cooled potatoes are less digestible than warm, cooked potatoes. It is vital to know the composition of the starch in potatoes and to choose proper processing and storage approaches to maximize the benefits. Starch digestion relies on its susceptibility to α-amylase and α-glucosidase (maltase) [20]. The digestion process is affected by the architecture of starch granules and the structure of glucan, as well as by the interaction between starch and other compounds [21]. Digestible starch is the major glycemic carbohydrate providing energy and is given as the first complementary nutrient in many areas [17].

Resistant starch (RS) is a form of starch and gets its name for resisting digestion in the small intestine [22], but it can be fermented by the colonic microflora in the large intestine [21,23]. RS as a functional ingredient normally derives from foods such as bananas, potatoes, grains, and legumes, acting as a substrate for the gut microbiome and providing a great many health benefits for human beings [18,23]. Indigestible starch was reported to enhance markers of bowel health, such as increasing short-chain fatty acid (SCFA) levels [22,24]. As Wolfsdorf and Weinstein (2003) reported, granular starches (uncooked starches) can be digested slowly and have the role of adjuvant therapy in some diseases, such as glycogen storage disease.

Potato starch, a B-type starch that has a smooth surface with no visible voids or pinholes [25], is classified as a type-2 resistant starch and is less likely to be vulnerable to digestive enzymes [26]. The starch granules do not dissolve in cold water before being modified (e.g., by cooking, chemical, or enzymatic modifications). Cooking with sufficient water initiates starch gelatinization, and the digestion of cooked starches is determined by the amylose content and amylopectin structure. The parameters of gelatinization vary with the source of the starch; some potato starches can be gelatinized at a temperature higher than 60 °C [17].

The glycemic index is an indicator of the digestibility and the production of glucose in starchy foods. A number of test methods are used to predict glycemic index in the body [27]. A disadvantage of blood glucose measurement is that blood sugar is buffered by glyconeogenesis, with the result that the amount of dietary glucose produced by starch cannot be accurately reflected [28]. To distinguish dietary glucose from total blood glucose, the isotope tracing methods of feeding ^13^C-labeled or ^13^C-enriched starchy materials were adopted [28].

The in vitro Englyst assay has been commonly applied in the food industry. This method classifies starches as “rapidly digestible starch (RDS)”, “slowly digestible starch (SDS)”, and “resistant starch (RS)”; this is debated by some researchers, such as Butterworth et al., who claims that all starch molecules are digested at the same rate and that there is no reason for the classification [29,30]. In addition, the Englyst assay does not take into account the interaction between starch molecules and mucosal α-glucosidases, both of which have different digestion mechanisms, from that of fungal glucoamylase [29].

Making good use of the starch in potato and properly preparing starchy foods are critical to clinical application. Starchy foods, especially amylopectin and modified starch, can be applied to young children’s diet as complementary feeding [31]. More recently, numerous works for health reason have been proposed, such as using potato peel powder as a protein/fiber source to make cakes. By observing the quality of the cake, the baking test showed that potato peel flour can significantly enhance the cake aspect and quality; in particular, by substituting wheat flour with 10% potato peel powder, cake with less hardness and a brighter and more saturated brown-orange color was obtained [32]. That study gave us a fascinating insight into and allowed us to discover a new way of developing fiber-rich cakes to supply dietary fiber intake. Additionally, as carbohydrates are an important part of a post-exercise recovery meal, potatoes can be consumed to fuel glycolysis during and after activity. Moreover, potato starch can be applied to manufacture polymers. Bio-friendly and natural-based materials are an innovative approach for plastic processing plants and packaging manufacturers.

### 2.3. Potato Starch and Edible Films

Starch, a natural polysaccharide, is one of the most commonly used materials for producing films or coatings. However, starch-based films are usually brittle and not resistant to water activity, which limits their application in packaging. To prepare starch-based films with good mechanical properties, water-resistant properties, as well as anti-microbial properties, numerous studies have been conducted. In particular, to protect and preserve fruits and vegetables, various starch-based composites (films or coating) were established, and their anti-microbial properties were assessed. Bajer et al. (2020) manufactured a novel biodegradable composite based on potato starch modified with chitosan, and aloe vera gel and glycerol as plasticizers were incorporated into the system. The composites showed good transparency, flexibility, as well as thermal resistance. The composite was found to be more susceptible to bacterial (*Bacillus* sp.) rather than fungal (*F. culmorum*) activity [33]. Zhang et al. (2019) suggested that the incorporation of mesoporous silica nanoparticles and cinnamon essential oil (CEO) into potato starch film markedly enhanced the tensile strength and physical properties of films, and the films also showed better antimicrobial activity against the FJ09 species than against the CNRMA 03.0371 strain [34].

By investigating the bio-composite films based on potato starch–glycerol–olive oil and incorporating zein nanoparticles (ZNP), Farajpour et al. reported that olive oil and zein nanoparticles can effectively reduce the water vapor permeability of the composite films, and that zein nanoparticles can increase their mechanical resistance [35]. The effects of different treatment methods such as pullulanase debranching (PD), ultrasound treatment (UT), and dimethyl sulfoxide heating (DSH) on the physicochemical properties of potato starch (PS) and PS-based films have been investigated. The results demonstrated that PS–LA composite films had higher tensile strength, lower elongation at break, and lower moisture permeability than native starch-based films, while films prepared by the PD method showed the highest tensile strength and lowest water vapor permeability in the tested films [36].

Some other researchers have used potato husk starch to prepare edible films. For example, edible films that were fabricated from by-products such as prickly pear peel mucilage (PPM) and potato husk starch (PHS) showed low water permeability (WP) [37]. Using agro-industrial wastes and exploring the applications in food packaging contributes to sustainable alternatives due to the recovery and reuse of the processing residues.

## 3. Potato Protein as Functional Food Ingredients

### 3.1. Potato Starch and Edible Films

Potato protein is reported to have a high biological value, which is measured according to the amount of protein from a specific food that can be applied to synthesize the protein in the organism. Some indicators can reveal the nutritional value of the protein, such as the essential amino acids index (EAAI) and the chemical score (CS) [38]. The CS makes a comparison of each essential amino acid (EAA) in the specific protein with the content of the standard protein, such as whole egg. The CS values of the EAAs and the EAAI can be calculated according to the reference protein of the joint FAO/WHO (1991).

### 3.2. Amino Acid Composition of Potato Protein

The EAAI and CS of EAAs present in potatoes of different flesh color varieties are shown in Table 1. Potato contains plenty of aspartic and glutamic acids and their amides as well as EAAs such as leucine, lysine, phenylalanine, valine, and tyrosine [39]. Potatoes are one of the best plant sources of lysine [40], an EAA generally absent in grains. The main fractions of proteins present in the tuber are patatin, protease inhibitors, and other high-molecular-weight proteins [41]. These are associated with several health benefits such as lower allergic response [42], antimicrobial effects [43], antioxidant potential [44], the regulation of blood pressure, blood serum cholesterol control [44,45], and anticarcinogenic behavior [46]. In comparison with egg white, potato protein is of great biological and nutritional value, and it has a CS range of 57–69 [47], which changes depending on the storage time and variety [38].

When the amino acid profile in dry matter of potato was checked, leucine was found to limit the quality of purple- and red-fleshed varieties while the sulfur amino acids methionine and cysteine primarily influence yellow-fleshed cultivars [38]. The results are consistent with the data reported by Eppendorfer et al. (1994), who thought that methionine, cysteine, and leucine were the most limited amino acids in the surveyed potato tubers [48]; however, this is in contrast to another report claiming that the sulfur amino acids were the limiting amino acids in all investigated potato varieties [49].

Peksa et al. surveyed the influence of storage conditions of potato on its amino acid composition, and they concluded that under certain conditions, the total protein and amino acid content showed a decreasing trend with increased storage time. The content of most amino acids decreased from 19 to 6% and from 38 to 21% after being stored for three and six months, respectively. Nevertheless, the coagulable protein showed a rising tendency and increased by 25%. The authors also claimed that storage temperature affected the coagulable protein content and serine, glycine, cysteine, tyrosine, and phenylalanine [38].

### 3.3. Fractions of Potato Protein

There are two key fractions of potato protein which can be considered to have high nutritive value: coagulable protein (nitrogen compounds which can be precipitated by trichloroacetic acid) and nonprotein organic compounds (such as free amino acids). In particular, coagulable protein is more valuable because of its well-balanced amino acid pattern [50]. The total and coagulable protein content in potatoes depends on the variety, not on the flesh color [38]. Different extraction techniques for tuber proteins such as precipitation methods (thermal and acidic precipitation, salt precipitation, ethanol precipitation, ammonium sulfate precipitation, carboxymethyl cellulose complexation), ion exchange, and separation of bioactive proteins and peptides have been explored to retain functionality and associated health benefits and, in turn, improve their potential application. The recovery of native protein from potato fruit water discharged from the starch factory has been commercially produced: in 2007, the Dutch Solanic subsidiary potato starch group of introduced a adsorbent processing platform with a mixed-mode ligand chemical method to extract high-performance proteins used in the food and pharmaceutical industries [51]. Jin et al. [52] studied the effect of the continuous polymer and pore phase known as Amberlite XAD7HP in lab-scale and pilot-scale experiments on the recovery of potato protein from potato fruit water; this method resulted in an extract primarily composed of a large proportion of protease inhibitors.

### 3.4. Potential Applications of Potato Protein

Cardiovascular disease (CVD), as a major global crisis, has been arousing wide interest around the world. The use of the angiotensin-converting enzyme (ACE) inhibitor to regulate blood pressure and cardiovascular function as a means to control hypertension and prevent CVD is currently considered to be very promising [53]. Even though several ACE inhibitors are currently available, we cannot ignore some of their side effects, such as dry cough and angioedema [54]. Thus, nutraceuticals such as bioactive peptides will be a choice to replace those drugs, which can reduce the cost of drug therapy and are safer [55,56]. Although a few doubts remain, some food-derived peptides have been shown to have ACE inhibitory activity in vitro, and many of them demonstrated significant anti-hypertensive activity in rat studies [57]. The arguments arise because of different study designs and diverse methods of measuring blood pressure, as well as a variety of genetic backgrounds of the study population [58]. Mäkinen et al. designed a schedule to check the effects of potato peptides (PP) and rapeseed peptides (RP) on blood pressure in vivo in the Goldblatt rat model of hypertension and showed that both peptides had anti-hypertensive effects due to the synergistic effects of several different ACE inhibitory peptide sequences in the samples [59]. According to current knowledge, more extensive research focus is needed on the practical application of food-derived bioactive peptides and on solving the issue of their insufficient effectiveness as a replacement for drug therapy in the actual treatment of hypertension. Meanwhile, other functional qualities of potato protein, such as their emulsification and foaming abilities, should be taken into account since they can help broaden the applications of potato in the food industry.

Potato protease inhibitors have a number of potential applications, such as treatment for obesity, perioral dermatitis, infections, cerebral thrombosis, and cancer. Previous research showed that potato protease inhibitors can promote the secretion of plasma cholecystokinin, which influences food intake [60,61]. This gastrointestinal hormone is related to satiety and food intake regulation as well as blood glucose control in humans, and its increased levels in plasma can delay the emptying of the stomach, resulting in feelings of fullness and reducing food intake. Most notably, Slendesta (a natural potato protein with 5% protease inhibitor P12) was found to enhance the feeling of fullness without any side effects. When food is eaten, a factor appears in the gut that is absorbed by the small intestine. The small intestine then releases a signal molecule called CCK into the bloodstream. CCK travels through the bloodstream to various digestive organs and to the vagus nerve, which signals the brain to produce feelings of fullness and satisfaction. A clinical study showed that it can help people manage hunger and thus achieve the goal of healthy weight management. Consuming 300 mg Slendesta (containing 15 mg P12) before dieting can have an effect on dietary intake since P12 helps to increase CCK levels and prolongs feelings of fullness [62,63,64]. Slendesta is produced by the American company KEMIN as a successful commercial product for controlling weight and provides new insight into solving obesity. Due to the potential sensitization of animal protein in susceptible populations, the use of plant-derived proteins as wine fining agents has received increasing attention.

Patatin P is the name of a family of glycoproteins which can be recycled from aqueous potato processing byproducts. It has been proved that patatin is an effective alternative for animal proteins, and it can be used as a fining agent since it can reduce total phenolics and tannins as well as reduce astringency and the content of red wine phenolics reacting with salivary proteins [65].

Potato tuber proteins can efficiently inhibit human fecal proteolytic activity, which makes it possible to prevent and treat this disease using potato protease inhibitors. Potato carboxypeptidase inhibitor has been considered as a potential fibrinolytic agent for the treatment and prevention of thrombotic disease since it can inhibit the plasma fibrinolysis inhibitor activated by carboxypeptidase and thrombin [66,67,68]. In addition, Blanco-Aparicio et al. (1998) showed that the potato carboxypeptidase inhibitor can compete with epidermal growth factor for binding to the receptor and has anti-cancer properties. The signal transduction pathway of the epidermal growth factor receptor plays a significant role in the development of neoplasms [46]. Potato tuber protease inhibitors can also affect the formation of hydrogen peroxide [69], which results from solar UV radiation [70]. Recently, a series of antimicrobial Kunitz-type serine protease inhibitors (AFP-J, PT-1, Potide-G) have been isolated from potato tubers. It has been shown that AFP-J displays potent antifungal activity against human fungal pathogens, e.g., Candida albicans, which is considered to be the main cause of candidiasis [71]. Furthermore, PT-1 can strongly inhibit various pathogenic microbial strains, including *C. Albicans*, the plant pathogenic fungus *Rhizoctoniasolani*, and the plant pathogenic bacterium *Clavibacter michiganese* [43]. Potide-G potently inhibited the growth of various human or plant pathogenic bacterial (*Staphylococcus aureus*, *Listeria monocytogenes*, *Escherichia coli*, *C. michiganense*) and fungal (*C. Albicans*, *R. solani*) strains, and also has potent antiviral activity against potato virus Y infection [72]. Based on current knowledge, more extensive research should focus on the practical application of food-derived bioactive peptides and on solving the issue of insufficient effectiveness to replace drug therapy in the actual treatment of hypertension. Meanwhile, other functional qualities of potato protein, such as emulsification and foaming abilities, should be taken into account since they can help broaden the application of potato in the food industry. Potato protease inhibitors have several potential applications involving treatment for weight loss, peri-anal dermatitis, infections, thrombotic disease, and cancer. Previous research showed that potato protease inhibitors can elevate plasma cholecystokinin levels which influences food intake [60,61]. This gastrointestinal hormone is involved in satiety and food intake regulation as well as blood glucose control in humans, and its increased levels in plasma delay gastric emptying, increase feelings of fullness, and reduce food intake.

## 4. Potato Phytochemicals and Nutritional Potential

### 4.1. Functional Phytochemicals in Potato

Potatoes are an excellent source of antioxidants, which include carotenoids, anthocyanin, phenolic compounds, and vitamin C. Nutritionally, these compounds play a role in preventing cancer and heart attacks with their potent antioxidative properties [73]. Carotenoids accumulate in many plants, giving yellow, orange, and red colors. The color of yellow potatoes is ascribed to carotenoids, which show high concentrations in yellow cultivars, while the color may be masked by anthocyanins in red- or purple-fleshed potatoes. *Carotenoids* contain primarily *lutein*, *zeaxanthin*, and *violaxanthin*, all of which are xanthophylls present in the flesh of potatoes. The composition of tuber carotenoid varies with cultivars; however, violaxanthin and lutein usually are the most abundant proportions.

### 4.2. Carotenoids and Health Benefits

The quantity of total carotenoids in vegetables varies among cultivars and ranges from 0.038 (potato) to 17.31 (spinach) [74] mg/100 g FW, whereas in potato, the value ranges from 0.038 to 2.00 mg/100 g FW [75,76,77]. Among all the fresh fruits and vegetables analyzed, certain potatoes have a comparable value (2.00 mg/100 g FW) [74] with other vegetables such as cabbage (0.25 to 0.43 mg/100 g FW) [74], strawberry (0.96 to 3.30 mg/100 g FW) [78], and tomato (1.63 to 8.57 mg/100 g FW) [79,80].

**Figure 1:**
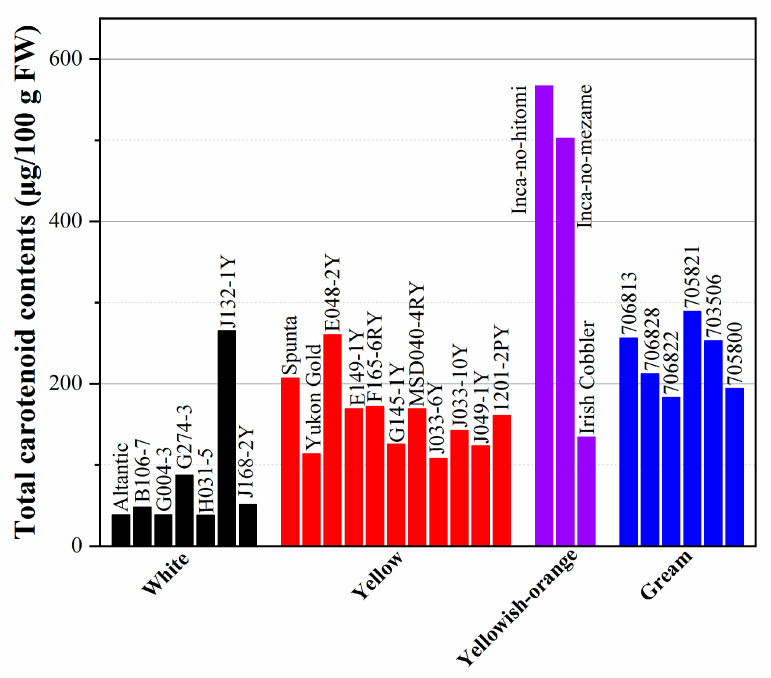
Total carotenoid concentrations by spectrophotometry and HPLC in potatoes.

The carotenoid concentration in colored flesh potatoes is shown in Figure 1. The content of carotenoids ranges from 38.1–265 μg/100 g FW in white-fleshed varieties and 107.5–260.3 μg/100g FW in yellow-fleshed cultivars to 567 μg/100g FW in yellowish–orange cultivars; some dark-yellow-fleshed cultivars even have a carotenoid content as high as 2000 μg/100 g FW (as shown in Figure 1). Among all the various colored fresh cultivars, yellow-fleshed varieties have the highest carotenoid content, followed by cream and white. Furthermore, over 100 cultivars grown in Ireland and Spain were shown to have carotenoid content from trace amounts to 28 μg/g DW in the skin and 9 μg/g DW in the flesh [81,82]. The lipophilic extract of potato with total carotenoids ranging from 35 to 795 per 100 g FW flesh shows 4.6–15.3 nmoles of α-tocopherol equivalents per 100 g FW of oxygen radical absorbance capacity (ORAC) values [83].

Furthermore, potato as a staple food is most commonly consumed and has the greatest daily intake every day compared with other selected vegetables. That is to say, potato can be a key and more accessible carotenoid supplement in our daily life. Potato is not the origin of pro-vitamins. However, carotenoids can have provitamin A activity and thus can decrease the risk of several diseases [84,85], age-related macular degeneration, and the onset of cataracts [86,87,88]. The process of carotenoid accumulation is the result of the biosynthesis, degradation, and stable storage of synthetic products [89,90].

Multiple factors control the broad diversity of carotenoid composition and content in storage tissue. Regulation of the catalytic activity of carotenoid biosynthesis can be a key and practical control for the final carotenoid accumulation. Phytoene synthase (PSY) is the rate-limiting step in the carotenoid biosynthetic pathway, and manipulation of PSY expression in many plants has been demonstrated to enhance carotenoid synthesis by directing metabolic flux into the carotenoid biosynthetic pathway [91,92,93].

**Figure 2:**
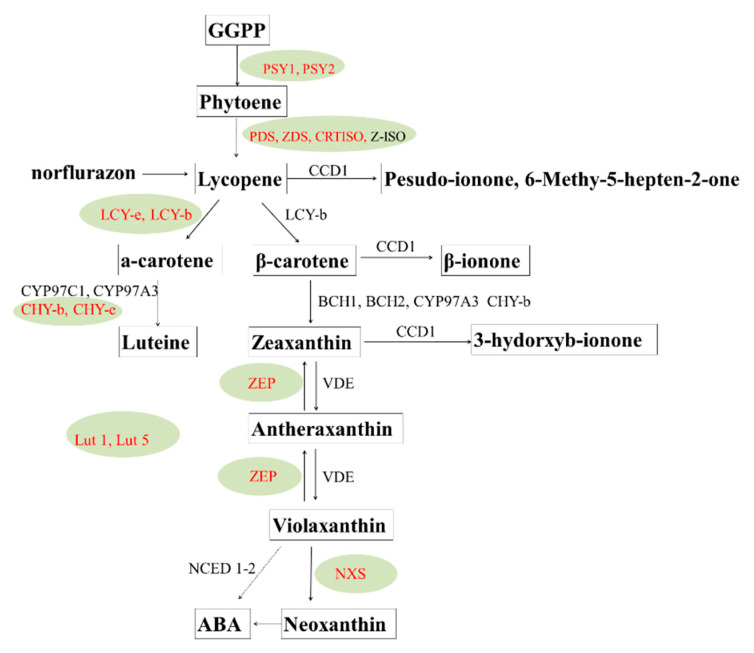
Biosynthesis, metabolic pathway. and gene regulation of carotenoid compounds in potatoes.

Carotenoid cleavage dioxygenases (CCDs) catabolize the enzymatic degradation of carotenoids. Expression of these genes inversely regulates carotenoid accumulation [94,95]. LCY-b and LCY-e can manipulate the synthesis of α-carotene and β-carotene, respectively. The tissue-specific expression of carotenoid biosynthesis genes in potato is marked in red color in Figure 2. Methods such as traditional breeding and metabolic engineering approaches have been utilized to improve the tuber carotenoid content. Traditional breeding can increase carotenoids based on broad-sense heritability of carotenoids [96]. The Y locus encodes a β-carotene hydroxylase and largely determines the tuber flesh color and zeaxanthin synthesis [97,98]. Molecular analysis has identified a QTL on chromosome 3 responsible for up to 71% of the carotenoid variation that is probably an allele of β-carotene hydroxylase, and several additional alleles affecting the amount of carotenoid have been identified [99,100]. A variety of transgenic approaches have achieved great success in increasing tuber carotenoids. The overexpression of bacterial phytoene synthase can increase total carotenoids from 5.6 to 35 μg/g DW, luteins 19-fold, and β-carotene from trace amounts to 11 μg/g DW [93]. Furthermore, a study showed that the overexpression of three bacterial genes in the Desiree potato caused a 3600-fold increase in β-carotene to 47 μg/g DW, a 30-fold increase in lutein, and a 20-fold increase in total carotenoids, producing a “golden potato” [101]. Manipulating the vitamin A pathway can fulfill 42% of the daily requirement for vitamin A (retinal activity equivalents) and 34% of the daily requirement for vitamin E by consuming a modest 150 g serving of boiled potatoes [102].

### 4.3. Phenolic Compounds and Antioxidant Activities

Potato supplies considerable phenolic compounds, which are concentrated in the peel and adjoining tissues [103]. The predominant one is chlorogenic acid (CGA), which consists of approximately 80% of the total phenolic acids. Red- and purple-fleshed potatoes usually contain more CGA than white potatoes. CGA may have a potential effect in reducing the risk of type 2-diabetes and slowing the entry of glucose into the bloodstream [104]. CGA in potatoes is synthesized via *hydroxycinnamoyl* CoA: *quinatehydroxycinnamoyltransferase* [105,106]. The R2R3 transcription factor StAN1 appears to mediate CGA expression and also regulates anthocyanins [107]. Phenylpropanoid (*phenolic acids*, *flavonols*, and *anthocyanins*) content varies markedly among cultivars, which is related in some way to the genetic diversity [108]. Andean potato landraces show about an 11-fold variation in phenolic acids and flavan-3-ols, and a high correlation between phenolics and total antioxidant activity [109,110,111]. Chilean landraces display 8- or 11-fold more phenylpropanoids than Desiree and Shepody, two common cultivars [112]. The phenolic content extracted from potato peel has been reported to have an antioxidant-mediated protective effect in erythrocytes against oxidative damage. However, polyphenol preservation is one of the keys to the quality of potato, affecting their flavor induction (astringency) and capacity to cause discolorations, such as enzymatic browning reactions [103]. Since most potato phenolics except anthocyanins are colorless, they present in white- and yellow-fleshed cultivars, which are desirable culinary ingredients in many countries. Total phenols/Total phenolic content (TPC) in a variety of plant cultivars as obtained from the Folin–Ciocalteau reagent (FCR) or HPLC was shown in Table 2.

Despite reviewing many research studies, it was difficult to make a comparison between various vegetables since different methods were used previously. Thus, this review summarizes the phenolic content in various plants as measured by the commonly used methods of Folin–Ciocalteau reagent (FCR) or HPLC and expressed relative to fresh weight and dry weight to provide a reference for next further study. By examining the total phenol of plants determined according to the Folin–Ciocalteau (F-C) colorimetric method and expressed on a gram of gallic acid equivalents (GAE) per kilogram of fresh weight basis (g GAE/kg FW), potato (0.31 to 8.83 g GAE/kg FW) is found to offer comparable value to certain vegetables and fruits, such as carrot (0.16 to 10.29 g GAE/kg FW) and blueberry (2.20 to 7.53 g GAE/kg FW), and is superior to some cultivars, such as cauliflower (0.57 to 2.55 g GAE/kg FW), cabbage (1.70 to 2.53 g GAE/kg FW) as well as strawberry (0.99 to 3.05 g GAE/kg FW). Furthermore, when expressed on a dry weight basis, potato (4.48 to 11.19 g GAE/kg DW) can provide almost the same quantity of total phenols as spinach. According to the maximum total phenol intake (based on a gram of gallic acid equivalents per kilogram of fresh weight) from plant cultivars, if 200 g potato were consumed every day, the total phenols from that potato would have to be provided by 700 g cabbage or cauliflower, 170 g carrot, 580 g strawberry, or 970 g mushroom; if 200 g potato were consumed every day, the total phenols from that potato ensures would have to be provided by 700 g cabbage or cauliflower, 170 g carrot, 580 g strawberry, or 970 g mushroom. On the other hand, according to the data obtained from the HPLC method, total phenols of potato range from 23.2 to 67.4 mg/100 g FW or 260–2852 mg/100 g DW, which are similar to those in the green bean (17.10 to 66.3 mg/100g FW). According to the data on the Singapore Chinese population aged 45–74 years obtained from the Singapore Chinese Health study from 1993 to 1998, the daily intake of potato is 6.9 g with 4.1 mg GAE/day of TPC. In conclusion, the potato should be a lower-cost alternative than other vegetables and fruits for daily total phenol intake; especially in some development areas, it can be the first consideration as an antioxidant source.

### 4.4. Flavonoids and Health Care Functions

Flavonoids in the flesh of white potato contain two leading constituents—catechin and epicatechin—and can be as high as 30 μg/100 g FW, almost twice that in red-and purple-fleshed potatoes [83]. One group suggested that *flavonols* increased up to 14 mg/100 g FW in fresh-cut tubers and suggested that they can be a valuable dietary source because of the large number of potatoes consumed [113]. The content of *flavonols* varies by more than 30-fold among different potato genotypes, and there is sizable variation even within the same genotypes. Interestingly, potato flowers can synthesize a 1000-fold higher amount of *flavonols* than tubers, which contain only micrograms per gram amount of *flavonols* [106]. Several studies show that quercetin and related *flavonols* have multiple health-promoting effects, including a reduced risk of heart disease; lower risk of certain respiratory diseases, such as asthma and bronchitis; and a reduced risk of some cancers, including prostate and lung cancer [114].

**Figure 3:**
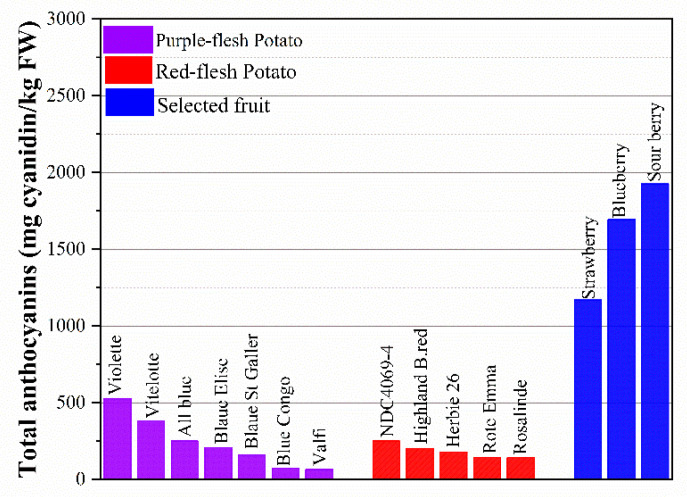
Total anthocyanins (mg cyanidin/kg FW) in selected fruits and potatoes.

Colored potatoes are rich in anthocyanins that exist abundantly in the skin of the potato or are partially or entirely derived from the flesh. Anthocyanins are natural colorants belonging to the flavonoid family [115]. These compounds are responsible for all the visible colors ranging from the red to blue of fruits, vegetables, flowers, and roots. Anthocyanidins commonly found in plants are delphinidin, cyanidin, petunidin, peonidin, pelargonidin, and malvidin [116]. Anthocyanin-rich foods have been shown to play an important role in the prevention of a wide range of human cancers, such as colon, breast, prostate, oral, and stomach cancers [117], while having no toxic effects on human somatic cells [5]. The total anthocyanin in selected fruits and potatoes is shown in Figure 3. Unpeeled potato with totally pigmented flesh can contain up to 527.4 mg/kg FW of total anthocyanins. Red or purple flesh with skin can be a profitable source of anthocyanins, similar to cranberries, and is superior to red cabbage [38]. As shown in Figure 3, the purple-fleshed potato has a higher total anthocyanin content than red-fleshed cultivars, ranging from 65.5 to 527.4 mg cyanidin/kg FW, while the value is 142–250 mg cyanidin/kg FW in red-fleshed potato. Compared with blueberry (1694 mg cyanidin/kg FW) and strawberry (1171 mg cyanidin/kg FW), which are known as high-anthocyanin foods, some varieties of potatoes are promising and have the potential to meet the demand for anthocyanins in daily life. What is most noteworthy is that potato is a more accessible food and is consumed in greater quantities than strawberry and blueberry since their high cost and seasonality limit the eating of those fruits. Through investigating over 50 colored-fleshed cultivars, researchers have found that anthocyanins vary in the range of 0.5–7 mg/FW in the skin and up to 2 mg/g FW in the flesh [118].

An issue that has come to our attention is that high-anthocyanin potatoes, usually with colored skin, are not as widely eaten as white or yellow potatoes. Tuber anthocyanin in the periderm is regulated by at least three loci—D, P, and R [119,120,121]. Biochemical and expression analyses revealed that an AN1 transcription factor complex was involved in potato anthocyanin synthesis [122,123], and its global expression in white and purple potatoes was studied using microarray and RNA-seq analysis. Multiple transcription factor variants were identified, including a ten amino acid C-terminal-modified AN1 required for optimal anthocyanin synthesis [124,125,126]. SSR markers for anthocyanin biosynthesis can be a technique for potato breeding programs [127]. Purple-fleshed potatoes known as a high-phenolic cultivar have been proved to benefit health by their anti-cancer properties [128,129,130,131] and amelioration of chromium toxicity [132]. In a mouse model, anthocyanins from purple potatoes were observed to show an effect of attenuating alcohol-induced hepatic injury [133]. In a human feeding study in which adults were fed 150 g of purple potatoes a day for six weeks, results showed that inflammation and DNA damage decreased [134]. Another small human trial suggested people with an average age of 54 years who consumed purple potatoes had a significant drop in blood pressure without any weight gain [135]. In addition, postprandial glycemia and insulinemia were observed to show a downward trend in males fed purple potatoes [135]. A previous study demonstrated that high polyphenol content in potato was inversely associated with their glycemic index [136]. For rats fed an obesity-promoting diet, purple potatoes promised metabolic and cardiovascular benefits [137].

### 4.5. Vitamins and Nutritional Potential

#### 4.5.1. Vitamin C

When it comes to the bioactive compounds in potato, we cannot ignore vitamin C, which has received the most attention. Vitamin C can be synthesized in the tuber part of the potato and transported to leaves and stems, where they then accumulate [138,139]. Potato generally contains 20 mg/100 g FW of vitamin C, which may account for up to 13% of the total antioxidant capacity. The recommended vitamin C intake per day for women (18–60 years old) is 60 mg. Potato (16.10 to 34.80 mg/100 g FW) [140,141] is a much better vitamin C source than cabbage (5.27 to 23.50 mg/100 g FW) [142] and even some kinds of tomato (8.26 to 22.54 mg/100 g FW) [143]. It should not be ignored as a crucial nutrient supplement based on vitamin C intake. Furthermore, in terms of the consumption rate and economical concerns, as well as storage conditions for vegetables, the potato could be the best choice for humans. As deficiency of iron has been a global problem, the consumption of potato with high vitamin C content might be a way to solve this problem.

Based on some research, the content of vitamin C is not only influenced by the potato variety, but also by the area and time of planting [144,145]. Much effort should be focused on maintaining vitamin C levels in cold storage since losses of up to 60% have been observed after cold storage [146,147]. By examining the vitamin C content in 12 potato genotypes after storing for 2, 4, and 7 months, a substantial loss after 4-month storage occurred, but several genotypes showed no significant loss after 2 months [148]. However, this contrasts markedly with a report that vitamin C increased as much as several-fold in 11 Indian potato varieties after storage [149]. It is highly desirable to find a cultivar that shows no loss after at least two months of storage. Other studies mentioned that compared with storage temperature, atmospheric oxygen levels have a greater effect on vitamin C levels [149].

Great care should be taken during processing because almost half the vitamin C is lost during pre-freezing, which can actually be avoided [150]. Therefore, optimizing processing and choosing proper methods to preserve crops is a practical measure to reduce the loss of vitamin C. Crop management also plays an important role in maximizing vitamin C, e.g., the use of high nitrogen fertilization reduces vitamin C levels, followed by a more rapid loss when the cut product is stored [151]. Consumers can choose potatoes with the peel to maximize phytonutrient intake. This theory proposes challenges for the food industry regarding how to improve the appearance and taste of the potatoes so that they can be widely accepted, as well as how to use the waste generated during processing and obtain more natural products, including antioxidants [152,153,154].

**Figure 4:**
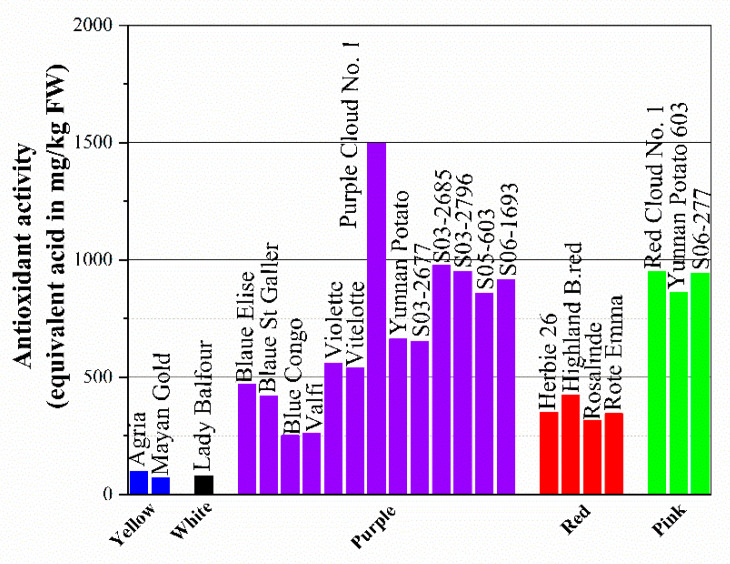
The total antioxidant activity (TAA) of potatoes was estimated using the ABTS radical cation method and DPPH assay.

According to some studies, the hydrophilic antioxidant activity of totally pigmented red or purple potato is comparable to Brussels sprouts or spinach. Total anthocyanins range from 9 to 38 mg/100 g FW and ORAC varies from 7.6 and 14.2 μmole/g FW of Trolox equivalents. Meanwhile, potato generally contains 20 mg/100 g FW of vitamin C, which may account for up to 13% of the total antioxidant capacity. The total antioxidant activity (TAA) of potatoes was estimated using the ABTS radical cation method, and the DPPH assay is presented in Figure 4. It can be concluded from the figure that purple-fleshed potato has the highest total antioxidant activity among the selected colors with a range of 251 to 1497.6 equivalent ascorbic acid in mg/kg FW, followed by pink (860.3–948.6 equivalent ascorbic acid in mg/kg FW) and red (316.9–424.4 equivalent ascorbic acid in mg/kg FW). This means that colorless cultivars, including white-fleshed ones and light-yellow-fleshed potato, generally have relatively low antioxidant activity, which might be responsible for the low content of vitamin C, anthocyanins, and even phenolic compounds.

Some other studies demonstrated a reverse effect of phytochemicals on various diseases (e.g., chronic inflammation, cardiovascular diseases, cancer, and diabetes) [155]. Many of the compounds discussed above are present in higher concentrations in immature potatoes. This is because certain tuber nutrient contents decrease with the growth of the tuber. Baby potatoes (of golf ball size) have amounts of phenylpropanoids as much as 3-fold that of mature potatoes of the same cultivar, and higher amounts of carotenoids and various other phytonutrients [156]. The CGA content decreased 39–72% during development and varied among cultivars [156].

By investigating the effects of domestic cooking methods (boiling, baking, steaming, microwaving, frying, stir-frying, and air-frying) on the composition of phytochemicals (phenolics, anthocyanins, and carotenoids) in purple-fleshed potatoes, a reduction in the *vitamin* C, total phenolic, anthocyanin, and carotenoid contents was observed after cooking. Among these changes, the decrease in antioxidant activity was responsible for a reduction in the total phenolic content. The loss of vitamin C and phytochemicals was caused by frying methods but did not alter the antioxidant activity, which is likely due to the prevention of by-products of the Maillard reactions. It should be noted that steaming and microwaving have the potential to be used as a measure to retain phytochemicals and antioxidant activity [157]. Xu et al. (2009) also concluded that all cooking methods (boiling, baking, and microwaving) cause a decrease in antioxidant activity and phytochemical concentrations in potato [158]. In contrast, according to the report of Blessington et al. (2010), baking, frying, and microwaving can significantly increase the total phenolic content, chlorogenic acid content, and antioxidant activity in potatoes [159]. Faller and Fialho (2009) also showed that despite a significant increase in the total phenolic content, the antioxidant activity of potatoes decreased [160], whereas Burgos et al. proposed that boiling has a positive effect in enhancing the total phenolic content and antioxidant activity and has an obvious reverse effect on the total anthocyanin content. These differences might be attributed to the different cultivars and pretreatment methods and cooking conditions, as well as the nonuniformity of the analytical methods used [161]. More importantly, anthocyanin usually is unstable and can be significantly influenced by different pHs [162]. Therefore, more systematic studies of the effects of processing and cooking methods on phytochemicals are needed, especially on the functions such phytochemical compounds have in various diseases.

#### 4.5.2. Vitamin B9

Folate (*vitamin* B9) deficiency is a worldwide concern that has a connection with birth defects [163]. Potatoes can be a source of dietary folate, providing 7–12% of the total folate in Dutch, Finnish, and Norwegian diets [164]. Increased consumption of potatoes can help to reduce the risk of low serum folate concentrations [165]. Folate concentrations in potatoes have been examined, which show that the content is significantly different among cultivars, with a range of 12–41 μg/100 g FW and 0.5–1.4 μg/g DW. Some wild species, such as S. Boliviense, contain 115 μg/100 g FW of folate [166,167,168].

#### 4.5.3. Vitamin B6

Vitamin B6 is indispensable in a wide range of metabolic, physiological, and developmental processes and shows a high concentration in potatoes. The USDA’s Supertracker website released a medium potato that can provide 48% of the supplement of vitamin B6. Vitamin B6 deficiency can contribute to numerous health issues, including diabetes and neurological and skin disorders. The maturity of the potato is related to the degree of vitamin B6 present. According to the report by Mooney et al., vitamin B6 ranged from 16 to 27 μg/g DW in immature and mature tubers, respectively [169]. There is also a small proportion of thiamine (vitamin B1) in potatoes, with a concentration of 0.06–0.23 μg/100 g FW [167,170]. Thiamine content shows moderate broad-sense heritability, suggesting that breeding programs can be the first consideration for increasing its levels [171].

## 5. Potato Minerals and Health Benefits

### 5.1. Potassium

Potato is also well known to be a provider of potassium, copper, phosphorous, iron, zinc, magnesium, and manganese. This makes it an indispensable candidate for contributing to the alleviation of mineral deficiencies caused by dietary constraints. In particular, as a potassium supplement, potato performs extremely well. The content of potassium in potatoes is higher than bananas, a well-known potassium source (SR28 2016). The potassium content increases during the entire growing season of potato [172]. The range of potassium content in potato is about 3.0–8.2 mg/g FW [14,173,174]. The content (mg/100 g) of certain macro-and microelements in edible parts of a variety of plant cultivars in comparison to recommended intakes is presented in Table 3. We conclude that potato, as a potassium source containing potassium in range of 104–540 mg/100 g FW, is superior to a number of vegetables and even has a comparable quantity with banana, which is known as a high-potassium food. Consumption of potato together with cabbage, cauliflower, spinach, or even cow milk can meet the recommended daily intake of potassium, which is about 3.1 g for an 18–60-year-old woman. In a study conducted in the Pacific Northwest, the potassium content for breeding lines and mature varieties was between 19–25 mg/g DW. The broad-sense heritabilities (H) of the Tri-State russet skin varieties and Regional Red/Specialty varieties were 0.33 and 0.81, respectively [175]. A survey of wild tuber-bearing Solanum species from the Commonwealth Potato Collection showed potassium levels between 15–27 mg/g DW [176].

The interaction between sodium and potassium can keep blood pressure in balance, reduce the risk of heart disease, strokes, and kidney stones, and ensure normal heart rhythms. The potassium in potatoes is also vital for helping muscles contract. Meanwhile, a study demonstrated that potassium deficiency correlates with the hardening of the arteries [177]. It is estimated that only 2% of Americans intake enough potassium. Potassium deficiency has been listed as a public health problem in the 2015–2020 Dietary Guidelines for Americans. The average adult intake is about half the 4700 mg/day recommended by the Institute of Medicine [178]. Storey and Anderson (2016) surveyed average nutrient intakes as well as total vegetable and white potato (WP) consumption among children aged 1–3 years using day 1 dietary data from the NHANES 2009–2012 and the Food Patterns Equivalents Database 2009–2012 and concluded that average intake of most nutrients, including calcium, exceeded the Dietary Reference Intakes (DRIs), but the average intake of potassium, dietary fiber (DF), and vitamin D was 67%, 55%, and 49% of DRIs, respectively. Finding excellent sources of potassium and DF, such as potatoes, is an urgent need today [179]. The final content of most minerals in potato relies on whether the skin is removed, and the method of processing, preparation, and cooking such material [14,180,181,182]. The USDA nutrient database typically lists the nutrient content of baked potatoes as within 5.5mg/g FW, while a potato that contains 8.2 mg/g FW would provide ~50% more potassium. Selecting the genetic capacity to produce higher amounts of potassium is expected to be on the agenda. The potassium in potato tuber increases throughout the growing season, as a report reveals [172]. Some factors such as the environment as well as crop management can affect the tuber potassium content; for example, drought conditions were found to increase K by about 12% [183].

### 5.2. Iron

Nearly two billion people worldwide suffer from iron deficiency, one of the most prevalent nutritional deficiencies according to the World Health Organization (WHO), which calls it a “public health condition of epidemic proportions” [184]. Potatoes contain moderate amounts of iron, which should be more easily bioavailable due to its lower phytic acid content than other crops. Considering that the broad-sense heritability estimate of potato is as high as 0.73, the development of new cultivars with higher iron content should be achievable [185,186]. A range of 17–62 μg/g DW was presented in a survey of potatoes grown in the Pacific Northwest, Colorado, and Texas [185]. Some varieties were reported to have a rather wider range of 0.3–2.3 mg Fe/g FW [187]. As shown in Table 3, the amount is 0.34–1.80 mg/100 g FW in potato. The iron content of some Andean germplasms is comparable to amounts in rice, maize, and wheat [188].

### 5.3. Magnesium

Magnesium is another mineral necessary for the human body. Several studies showed that increased magnesium intake may help to inhibit diabetes, one of the most widespread and costly diseases [189]. The RDA for adults ranges from 310 to 420 mg. It is estimated that nearly 70% of American adults lack Mg, and another 20% of them consume less than half of the recommended amount of magnesium. Mg deficiency is associated with higher levels of inflammation and cardiovascular risk [190]. Potato magnesium levels were reported to range from 142 to 370 μg/g FW [191,192] and 0.9–1.1 mg/g DW. However, the magnesium content in potato shown in Table 3 is 13.7–41.9 mg/100 g FW. This content is almost equal to the quantities in cabbage. The cultivated area of potato could be a factor that decides the content of Mg. In the Pacific Northwest, Colorado, and Texas, the magnesium content of potatoes ranged from 787 to 1089 μg/g DW [193]. In the Tri-State and Western Regional russet skin trials and the Red/Specialty trials, the broad heritability (H) was 0.57, 0, and 0.72, respectively. Crop nutrient management was reported to have different effects on Mg. As showed, it can affect Mg more than K [194], while another study found little effect of fertilizer on Mg. In addition to the minerals discussed above, some other minerals in potato were also examined [195,196,197].

### 5.4. Phosphorus

Other than potassium, there is another main mineral present in the tuber—phosphorus. It plays a crucial role in the human body. Inadequate phosphorous intake results in abnormally low serum phosphate levels, which result in loss of appetite, anemia, loss of muscle, tingling of extremities, and difficulty walking. The daily requirement for phosphorus is 800–1000 mg. In potatoes, phosphorus ranges from ~1300 to 6000 µg/g DW [172,174,196] and 30–60 mg/100 g FW (shown in Table 3). A study reported that the fertilizer P applied together with nitrogen resulted in increased phosphorus in potato parts but without a significant impact on the yield of tubers [198]. However, potatoes show a combination of high P requirement with low P uptake [199]; thus, a large amount of P is needed to plant potatoes. In addition to improved management practices to reduce P losses to the environment and to close nutrient cycles, new cultivars with higher P efficiencies may contribute to a more sustainable use of P resources.

### 5.5. Acrylamide

Acrylamide is a kind of carcinogenic substance, which is formed from the Maillard reaction between asparagine and reducing sugars [200,201]. Acrylamide in potato products is formed primarily during the high-temperature frying process prior to factory processing and retail [202]. Fried potato chips contain as much as 4.18 mg/kg acrylamide [203]. The European Food Safety Authority announced the acrylamide content monitoring data of foods in 2012 [203]. Several possible methods to reduce acrylamide content have been reported in the literature, including the selection of potato varieties with low reducing sugar content and the storage of potatoes in hypothermal storage cellars [204]. Adding asparaginase is another method to reduce acrylamide content. Asparaginase can transform the main precursor of acrylamide into aspartate, thus controlling the production of acrylamide in foods. This method can reduce the content of acrylamide to below the detection limit in French fries [205]. In addition, frying with vacuum pressure is another effective way to reduce acrylamide content. Yagua et al. [206] reported that this method can effectively reduce acrylamide content by 94%, because vacuum frying can be performed at very low temperatures.

## 6. Conclusions

As the story of potatoes in the human diet continues to evolve, new and advanced techniques will promise significant opportunities to further explore the role of potatoes and their functional properties. Both in Asia and Africa, potato production has boomed, especially in China, where potato production is by far the largest. China has made potatoes its forward-looking food security strategy, whereas the production in the West has declined. With the characteristics of ease of cultivation, minimum transportation and storage conditions, and being rich in carbohydrates, vitamins, and antioxidants, potato as a staple food still has much scope to be examined. Remarkably, potato contributes only 440 calories per pound, which makes it a promising healthy food source to meet today’s requirements of dieters. Potato starch has some unique properties compared with cereal starches. The most significant ones involve long amylopectin chains forming hydrated and ordered B-type starch with orthorhombic crystalline structures. Thus, more efforts should focus on the actual model of the interconnection of cluster units of the amylopectin component and the connection between the proposed orthorhombic crystalline structure and the hexagonal crystalline structure in the granules. Genetic engineering can also be a possible solution to help to control the synthesis and development of potato starches by altering structures and functionality. Owing to their high consumption, potato proteins are of particular interest, and nutritionally, they are superior to the animal protein Iysozyme. Different extraction techniques for tuber proteins such as precipitation methods (thermal and acidic precipitation, salt precipitation, ethanol precipitation, ammonium sulfate precipitation, carboxymethyl cellulose complexation), ion exchange, and separation of bioactive proteins and peptides have been explored to retain their functionality and associated health benefits and, in turn, improve their potential application. Moreover, the potato has a complex chemical composition that includes important amounts of minerals, vitamins, and phytonutrients. To fight against the assertion of some scientists that the carbonates in potato are the primary contributor to obesity, continued research should be conducted on the next step. While considering increasing the phytonutrient content of potato, advances in crop management work together with traditional breeding, and molecular breeding such as the use of TALEN and CRISPR can be a potential option. The strong incentive for quality control and the safety of final products calls for applicable methods to analyze biologically active alkaloids and glycoalkaloids in potato. Accurate analyses of these controversial components will also benefit the grower, processor, researcher, and consumers. Further studies such as studies of toxicities and beneficial health effects as well as investigation of the biosynthesis of the secondary metabolites are needed. Additionally, knowledge of the impact of climate change, genetics, and reproductive dynamics of potato, as well as purification of waste streams (both the fruit and the pulp), will be essential topics for researchers around the world. Advances in genomics, proteomics, metabolomics, and bioinformatics will open a door to produce new generations of advanced germplasms for emerging priorities or special needs. In recent years, there has been increasing interest in finding functional foods, such as the natural antioxidants which can protect the human body from radicals and retard the process of many chronic diseases, and special proteins, as well as fiber functions with healthy and industrial additives.

## Figures and Tables

**Table 1:** Essential amino acid index (EAAI) and chemical scores of essential amino acids present in potatoes of different flesh color varieties.

Essential Amino Acids					Limiting Amino Acids						
Variety	EAAI	Thr	Met + Cys	Val	Ile	Leu	Phe + Tyr	Lys	∑eaa	1st	2nd
Blaue Annelise	79	71	63	93	92	63	118	65	81	Met + Cys	Lys + Leu
Blue Congo	86	76	59	114	102	63	134	79	91	Met + Cys	Leu
Herbie 26	96	82	78	125	112	72	134	88	79	Leu	Met + Cys
Rote Emma	110	93	78	146	129	88	165	101	116	Met + Cys	Leu
Vineta	82	82	63	103	89	63	115	75	85	Met + Cys	Lys + Leu
Fresco	118	115	92	154	122	84	182	102	123	Leu	Met + Cys
Protein standard (g/16 g N)		3.4	2.5	3.5	2.8	6.6	6.3	5.8	30.9	-	-

Note: Adapted from [38].

**Table 2:** Total phenols in a variety of plant cultivars as obtained from the Folin–Ciocalteau reagent (FCR) or HPLC.

Variety/Plant Cultivar	FCR		HPLC	
	g GAE/kg FW	g GAE/kg DW	mg/100 g FW	mg/100 g DW
Cauliflower	0.57–2.55	5.60–29.13		30–217
Cabbage	1.70–2.53	1.90–26.29	0.50	125–387
Broccoli	0.96–3.76		1.10–1.14	1173
Spinach	0.50–2.34	11.8		
Carrot (orange & black)	0.16–10.29	1.10–1.90	0.30–39.76	
Brinjal	0.82–2.92			
Green bean	0.78–4.58		17.10–66.3	
Mushroom	0.09–1.80			
Tomato	0.14–2.91		26.57	
Strawberry	0.99–3.05			
Blueberry	2.20–7.53			
Kiwifruit	0.80–0.96			
Pea		0.80–1.20		
Brussels sprouts				147
Italian kale				1127
Potato	0.31–8.83	4.48–11.19	23.2–67.4	260–2852

**Table 3:** Content (mg/100 g) of certain macro- and microelements in edible parts of a variety of plant cultivars in comparison to recommended intakes (mg/100 g FW).

	Macro-Elements				Micro-Elements	
	K	Ca	Mg	P	Fe	Zn
Recommended intake/day	3.1 g	800 mg	280 mg		12–18 mg	7 mg
Banana	261–546	0.35~1.35	80.0~84.0	/	17.15~47.19	0.34
Cauliflower	389–705	6.33	5.00	/	0.29	0.12
Broccoli	405–727	8.87–11.40	5.9	/	0.26	0.87
Spinach	210–630	98.0–241.39	11.1–85.0	/	3.90–9.49	0.2–0.55
Pepper	159–244	8.8–13.9	11.2–18.8	17.6–37.6	288–975	270–775
Cabbage	165–340	70–152	12.0–42.0	32	1.4–1.7	0.3–0.5
Carrot	357	25	71.0	/	9.28	1.28
Strawberry	153–312	7.6–29	11.0–23.4	0.5–24	0.20–0.62	0.14–1.5
Cow milk	152	122	12.0	119	0.08	0.530
Kiwifruit	254–300	15.4–26	11.0–13.1	24–25	0.19–0.25	0.09–0.14
Potato	104–540	4.98–21.29	13.7–41.9	30–60	0.34–1.80	0.27–0.53

## Data Availability

Data is contained within the article.
